# Smartphone App–Guided Pulmonary Rehabilitation in Chronic Respiratory Diseases: Randomized Controlled Trial

**DOI:** 10.2196/64884

**Published:** 2025-08-25

**Authors:** Chiwook Chung, Deog Kyeom Kim, Jung-Kyu Lee, Eun Young Heo, Hee Kwon, Dongbum Kim, Woo Jin Kim, Sei Won Lee

**Affiliations:** 1 Division of Pulmonary, Allergy and Critical Care Medicine, Department of Internal Medicine, Hallym University Dongtan Sacred Heart Hospital, Hallym University College of Medicine, Hwaseong, Republic of Korea; 2 Department of Pulmonary and Critical Care Medicine, Asan Medical Center, University of Ulsan College of Medicine, 88 Olympic-ro 43-gil, Songpa-gu, Seoul, 05505, Republic of Korea, 82 2-3010-3990, 82 2-3010-6968; 3 Division of Pulmonary and Critical Care Medicine, Department of Internal Medicine, Seoul National University College of Medicine, Seoul Metropolitan Government Boramae Medical Center, Seoul, Republic of Korea; 4 LifeSemantics Corp, Seoul, Republic of Korea; 5 Department of Internal Medicine and Environmental Health Center, Kangwon National University, Chuncheon, Republic of Korea

**Keywords:** pulmonary rehabilitation, chronic respiratory diseases, COPD, lungs, respiratory, mHealth, apps, smartphones, walking, randomized controlled trial

## Abstract

**Background:**

Pulmonary rehabilitation improves exercise capacity, dyspnea, quality of life, and survival in patients with chronic respiratory disease. However, center-based pulmonary rehabilitation programs remain unavailable in many health care facilities due to several barriers. To address this, we developed a smartphone app that enabled individuals to perform pulmonary rehabilitation at home.

**Objective:**

We aimed to evaluate the efficacy of smartphone app–guided pulmonary rehabilitation in improving exercise capacity in individuals with chronic respiratory diseases.

**Methods:**

This was a multicenter prospective, single-blind, randomized controlled trial conducted in 2022. A total of 100 participants with chronic respiratory disease, including chronic obstructive pulmonary disease, asthma, and lung cancer, were recruited, with equal distribution (50:50) between the intervention group and the control group. The intervention group followed a 12-week app-guided rehabilitation program, while the control group received standard outpatient treatment. The primary outcome was the 6-minute walk test distance (6MWD) after the 12-week rehabilitation period. Secondary outcomes included quality of life questionnaires and health care usage.

**Results:**

Among the 100 participants included, 88 completed the follow-up visit (41 in the intervention group and 47 in the control group). Their median age was 68.0 years, and 72 (81.8%) were men. Most participants (n=70, 79.5%) had a smoking history, with a median of 40.0 pack-years. Their forced expiratory volume in 1 second was a median of 63.0% (IQR 50.5‐71.5). Most participants (n=85, 96.6%) had chronic obstructive pulmonary disease. After the 12-week rehabilitation program, 6MWD was not different between the intervention and control group (median 490.0, IQR 468.8‐556.3 vs 485.0, IQR 440.0‐527.3 m). Assuming a clinically minimal effective change of 25 meters in 6MWD, only 7 out of 41 participants among the intervention group achieved the minimal clinically important differences after the rehabilitation program. Quality of life questionnaire scores, including the St George’s Respiratory Questionnaire and Hospital Anxiety and Depression Scale, did not differ between groups. In addition, none of the participants experienced hospitalization or emergency room visits during the study period. Regarding the service satisfaction questionnaire, more than 3-quarters of the intervention group (34/41) rated their scores as ≥17/20.

**Conclusions:**

In this study, smartphone app–guided pulmonary rehabilitation failed to improve exercise capacity and quality of life in patients with chronic respiratory diseases. However, the results indicated that older adults with chronic respiratory conditions can safely use smartphone app–guided pulmonary rehabilitation. Thus, smartphone app–guided pulmonary rehabilitation may be a feasible option for older adults with chronic respiratory disease.

## Introduction

Chronic respiratory diseases are significant contributors to global mortality and morbidity [1,2]. In 2019, chronic obstructive lung disease (COPD), lung cancer, and lower respiratory infections ranked among the top 10 leading causes of disability-adjusted life years for adults aged 50 years and older [1]. Individuals with chronic respiratory diseases experience various clinical deteriorations, including poor quality of life and reduced exercise capacity [3,4]. Previous studies have shown that lower exercise capacity and physical activity levels are associated with higher risks of exacerbation and mortality in individuals with COPD [5,6]. Exercise capacity and physical activity serve as independent predictors of mortality in individuals with advanced lung cancer [7].

Pulmonary rehabilitation, a comprehensive intervention, aims to enhance the physical and psychological well-being of individuals with chronic respiratory disease through exercise training, education, and behavior change [4]. Pulmonary rehabilitation increased exercise capacity, improved quality of life, and reduced dyspnea in individuals with chronic respiratory diseases [3,4,8]. Previous studies have highlighted that muscle wasting and dysfunction, known as respiratory sarcopenia, were commonly found in individuals with chronic respiratory diseases, which is associated with decreased exercise capacity and respiratory function [9,10]. By combining pulmonary rehabilitation with nutritional management, we can potentially prevent and treat respiratory sarcopenia and further enhance respiratory function [10].

Previous landmark studies presented pulmonary rehabilitation programs that include exercise training lasting 30‐45 minutes per day, 3‐5 days per week, and at least 8‐12 weeks [11,12]. However, these studies also highlighted challenges associated with center-based pulmonary rehabilitation, such as limited facilities, transportation barriers, low social support, and lack of motivation [13,14]. Furthermore, the COVID-19 pandemic necessitated alternatives to face-to-face center-based rehabilitation for individuals with chronic respiratory diseases [15]. Consequently, we designed a pulmonary rehabilitation program and developed a smartphone app called Redpill Breath (LifeSemantics), enabling individuals to perform pulmonary rehabilitation at home. Our study aimed to evaluate the efficacy of smartphone app–guided pulmonary rehabilitation in improving exercise capacity in individuals with chronic respiratory diseases.

## Methods

### Study Design

This multicenter prospective, single-blind, randomized controlled trial aimed to evaluate the clinical effectiveness and safety of pulmonary rehabilitation using the smartphone app Redpill Breath for patients with chronic respiratory disease, including COPD, asthma, and lung cancer. In 2022, a total of 100 participants (50 in the intervention group and 50 in the control group) were recruited from the outpatient pulmonology clinics at Asan Medical Center (Seoul, Republic of Korea), Kangwon National University Hospital (Chuncheon, Republic of Korea), and Seoul Metropolitan Government Seoul National University Boramae Medical Center (Seoul, Republic of Korea). Participants were recruited from each health care center through poster advertisements from January 2022 to September 2022. The trial started in January 2022 and finished in November 2022. Eligible participants were interviewed by trained clinical research coordinators to confirm their eligibility. After the eligibility screening, participants were randomly assigned to either the intervention or control group using a prepared randomization sequence.

At the time of enrollment, clinical research coordinators collected participants’ baseline information, including age, sex, height, weight, BMI, smoking history, and underlying comorbidities. The study outcomes were measured twice, before and after the 12-week rehabilitation program. In addition, the intervention group completed a service satisfaction questionnaire after the end of the study. The intervention group participated in a 12-week app–guided rehabilitation program. They were equipped with a Bluetooth-connected wrist pulse oximeter (either the WristOx 2 3150, Nonin, or the MD300W628, ChoiceMMed) to monitor heart rate and oxygen saturation during exercise. In contrast, the control group received standard outpatient treatment, which included medical treatment using bronchodilator inhalers according to current guidelines, along with brief advice on the importance of physical activity. They were also provided with a leaflet containing instructions on pulmonary rehabilitation (center-based rehabilitation programs were not provided). The leaflet provided static illustrations and text-based instructions for walking exercise, muscle training, and an exercise diary [16]. In contrast, the app provided interactive guidance, video demonstrations, progress tracking, and motivational messages, offering a more engaging and personalized experience. This study was conducted in line with the CONSORT-EHEALTH (Consolidated Standards of Reporting Trials of Electronic and Mobile Health Applications and Online Telehealth) guidelines (the CONSORT-EHEALTH checklist is provided in Checklist 1).

### Inclusion and Exclusion Criteria

The inclusion criteria for this study were as follows: (1) participants aged 19-80 years; (2) presence of dyspnea symptoms (at least 1 point on the modified Medical Research Council Dyspnea Scale [mMRC]) and impaired lung function, defined as [1] either forced vital capacity (FVC) or forced expiratory volume in 1 second (FEV1) <80% predicted for patients with lung cancer or [2] postbronchodilator FEV1/FVC <0.7 and postbronchodilator FEV1 <80% predicted for patients with other respiratory disease; (3) ownership of an Android smartphone (OS 8.0 or higher, with internal memory of 32 GB or higher) or an iPhone (iOS 13.0 or higher, with internal memory of 32 GB or higher); (4) ability to effectively use a smartphone and mobile software, and willingness to bring their smartphone for a walking test during the 12-week clinical trial; and (5) voluntary informed consent.

The exclusion criteria were as follows: (1) respiratory distress syndrome caused by neuromuscular diseases, spinal cord injury, or thoracic deformities; (2) participation in pulmonary rehabilitation treatment within 6 months of the screening date; (3) disease exacerbation requiring antibiotics or steroid treatment within 2 weeks of the screening date; (4) unstable cardiovascular diseases (such as unstable angina pectoris, acute myocardial infarction, or severe coarctation of aorta); (5) uncontrolled pulmonary arterial hypertension; (6) physical disabilities or cognitive impairments; (7) pregnancy or breastfeeding; (8) illiteracy; (9) participation in other clinical trials within 90 days of the screening date; and (10) deemed inappropriate to participate based on the clinical research associate’s opinion.

### Smartphone App and Rehabilitation Program

**Figure 1.**
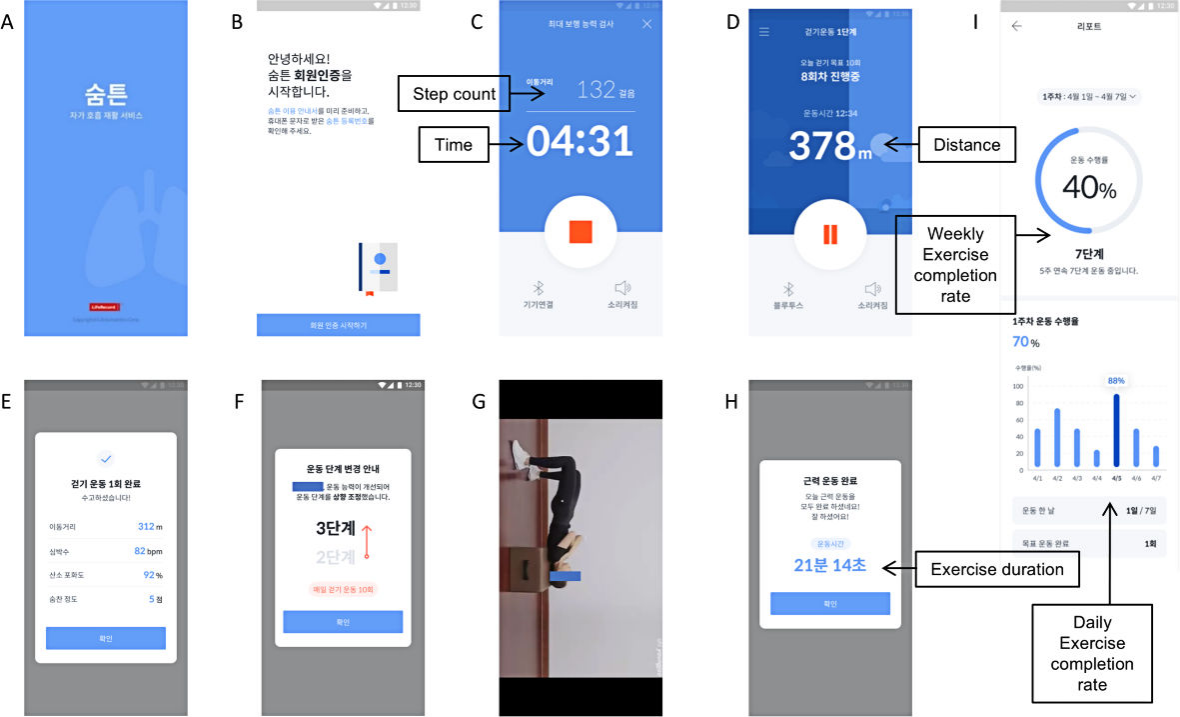
Screenshots of the Redpill Breath app. Shown are the following: (**A**) opening screen; (**B**) account creation; (**C**) the 6-minute walk test, which displays step counts and test time; (**D**) walking distance and timer for walking exercises; (**E**) completion of walking exercise, which displays walking distance, heart rate, oxygen saturation, and rating of perceived exertion scale evaluated after exercise; (**F**) exercise level change; (**G**) instruction videos for muscle training exercise program; (**H**) completion of muscle training exercise; and (**I**) exercise report, which displays exercise completion rate per week and day.

The smartphone app Redpill Breath was developed collaboratively by the investigators and LifeSemantics Corp. Available on both Android and iPhone platforms (requiring at least Android 8.0 or iOS 13.0), the app incorporates an exercise program based on established guidelines [3,17,18]. Key features include a 6-minute walk test (6MWT), a daily walking exercise regimen (25‐35 minutes) combined with muscle training (20‐25 minutes), exercise records, and real-time heart rate and oxygen saturation monitoring during walks. Users benefit from audio guidance for walking exercises and video instruction for muscle training. In addition, the exercise program’s intensity can be tailored based on the 6-minute walk test distance (6MWD; Figure 1A-I).

### Study Outcome

The original study protocol was designed to measure the study outcomes twice, at 8 and 12 weeks after the baseline. However, this was modified to assess the outcomes only once, 12 weeks after the baseline. The primary outcome measure was the 6MWD after the 12-week rehabilitation program. The test was conducted on a 30-meter track using previously described methods [19]. Secondary outcomes included assessments using the mMRC dyspnea scale, the St. George’s Respiratory Questionnaire (SGRQ) [20], the Hospital Anxiety and Depression Scale (HADS) [21], as well as tracking hospitalizations, emergency room visits, and a service satisfaction questionnaire (Patient Global Assessment, applicable to the intervention group only). The satisfaction questionnaire consisted of 4 questions with scores ranging from 1 to 5 for each question (total score range 4‐20). Within the intervention group, participants with compliance >70% were categorized into the per-protocol analysis set (PP set) according to the study protocol.

### Randomization and Sample Size Calculation

Participants were evenly divided into the intervention and control groups at a 1:1 ratio (50 participants in each group). Upon enrollment, participants were randomly assigned to either group before undergoing baseline tests, and the randomization sequence was concealed until the time of randomization. The random sequence was generated using stratified block randomization with the Proc plan procedure in SAS software version 9.4 (SAS Institute).

The sample size estimation was based on the minimal important difference in the 6MWD observed in previous studies [22-24]. We assumed a 25-meter difference in 6MWD between the groups and an SD of 52.5 meters. With an alpha level of .05 and a power of 80%, to achieve a 95% CI with the difference between groups >0 meters, we needed a minimum of 40 participants in each group. To account for a potential dropout rate of 20%, we aimed for 100 participants (50 in each group).

### Statistical Analysis

Continuous variables were reported as median (IQR) and compared using the Mann-Whitney *U* test. Categorical variables were presented as numbers (percentages) and compared using the chi-square or Fisher exact test. All *P* values were 2-tailed, with statistical significance set at *P*<.05. The statistical analyses were conducted using MedCalc Statistical Software version 22.021 (MedCalc Software Ltd).

### Ethical Considerations

The study protocol was approved by the institutional review board of each hospital (Asan Medical Center: 2021‐1108, Kangwon National University Hospital: A-2021-07-009, and Seoul Metropolitan Government Seoul National University Boramae Medical Center: 30-2021-103). Written informed consent was obtained from all participants prior to inclusion. The study complied with the guidelines stipulated in the Declaration of Helsinki, and all methods were performed in accordance with the relevant guidelines. This study was registered in the ClinicalTrials.gov database (NCT05299385). Data were deidentified and participants did not receive compensation.

## Results

### Participant Baseline Characteristics

**Figure 2.**
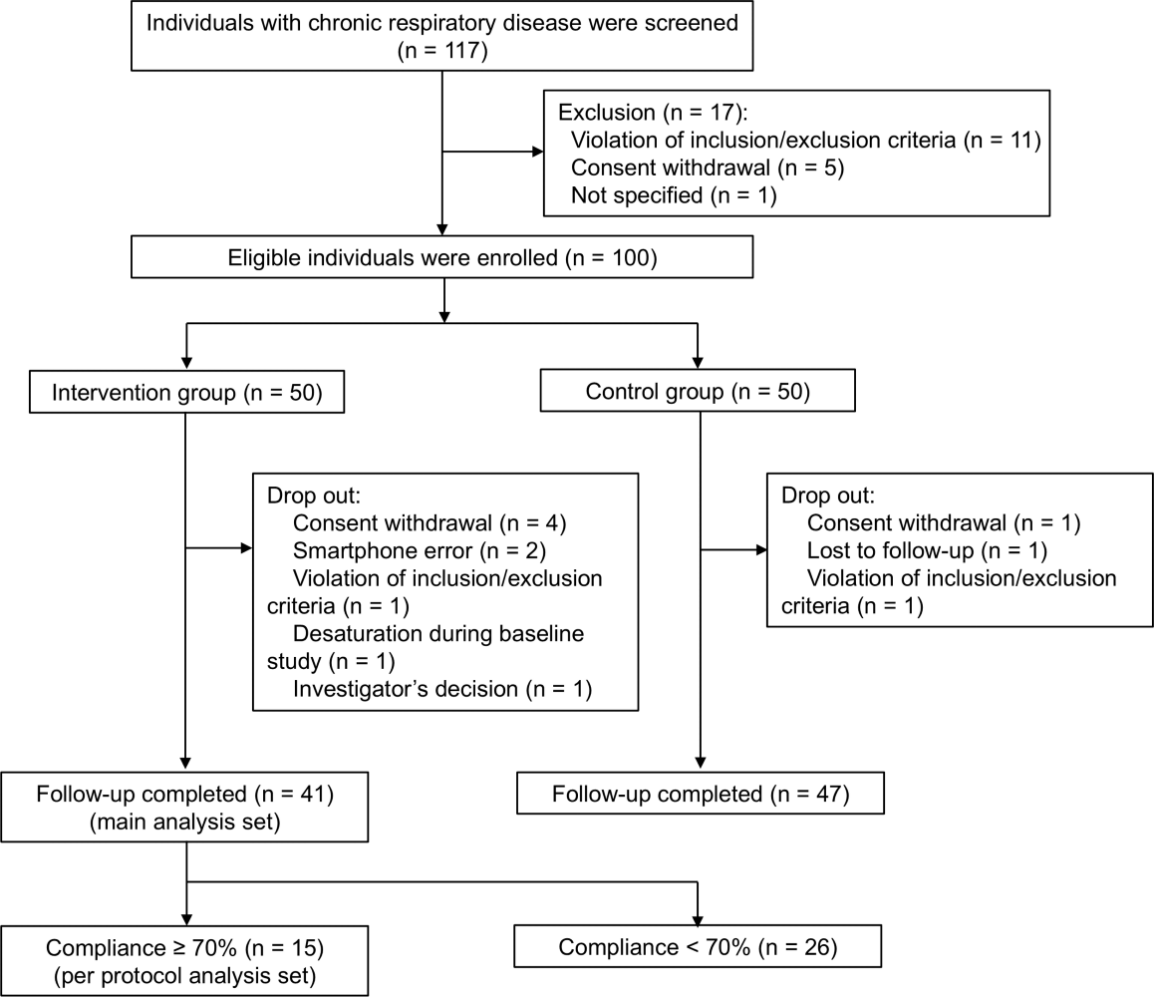
Study flowchart.

Figure 2 illustrates the study process. Out of 117 individuals screened, 100 were eligible and included in the study. In the intervention group, 9 participants dropped out, and 41 completed the follow-up visit. Among them, 15 achieved exercise program compliance of ≥70% and were categorized into the per-protocol analysis set as follows: 0%‐20% compliance (11 participants), 21%‐40% (8 participants), 41%‐60% (5 participants), 61%‐80% (3 participants), and 81%‐100% (14 participants). In the control group, 3 participants dropped out, and 47 completed the follow-up visit. No participants experienced musculoskeletal injury related to the rehabilitation program during the study period.

Table 1 presents the baseline characteristics of the study participants. Their median age was 68.0 (IQR 62.0‐73.0) years, and 72 (81.8%) were men. Most of the participants (n=85, 96.6%) had COPD as their respiratory condition, with a median disease duration of 4.9 years. Furthermore, 70 (79.5%) participants had a smoking history, with a median of 40.0 pack-years. Their FEV1 was reduced, with a median of 63.0% (IQR 50.5%‐71.5%).

**Table 1.** Baseline characteristics of study participants.

Variables	Total (n=88)	Intervention (n=41)	Control (n=47)	*P* value
Age (years), median (IQR)	68.0 (62.0‐73.0)	67.0 (59.0‐71.0)	69.0 (63.3‐73.0)	.10
Male sex, n (%)	72 (81.8)	36 (87.8)	36 (76.6)	.14
Height (cm), median (IQR)	165.0 (159.1‐169.0)	166.0 (159.1‐170.1)	164.0 (159.3‐168.0)	.28
Body weight (kg), median (IQR)	65.0 (56.0‐73.0)	67.0 (56.5‐77.0)	64.0 (6.0‐71.0)	.25
BMI (kg/m^2^), median (IQR)	24.1 (21.7‐26.7)	24.9 (21.9‐27.5)	24.0 (21.7‐26.4)	.56
Respiratory disease, n (%)				.26
COPD^a^	85 (96.6)	41 (100.0)	44 (93.6)	
Asthma	2 (2.3)	0 (0.0)	2 (4.3)	
Bronchiectasis	1 (1.1)	0 (0.0)	1 (2.1)	
Duration of disease (years), median (IQR)	4.9 (2.1‐9.7)	4.8 (1.9‐9.4)	5.0 (2.2‐10.1)	.51
Ever-smokers, n (%)	70 (79.5)	34 (82.9)	36 (76.6)	.47
Amount, pack-years (n=70), median (IQR)	40.0 (20.0‐45.0)	35.0 (22.5‐45.0)	40.0 (20.0‐45.0)	.72
Lung function, median (IQR)				
FEV1^b^ (% predicted)	63.0 (50.5‐71.5)	63.0 (53.0‐71.3)	63.0 (48.3‐71.8)	.98
FVC^c^ (% predicted)	83.0 (73.5‐88.5)	84.0 (75.5‐89.3)	81.0 (73.0‐87.8)	.57
FEV1/FVC	0.54 (0.44‐0.62)	0.54 (0.45‐0.62)	0.54 (0.43‐0.64)	.88

a COPD: chronic obstructive pulmonary disease.

b FEV1: forced expiratory volume in one second.

c FVC: forced vital capacity.

### Clinical Parameters of Participants

Analysis of the clinical outcomes at follow-up indicated no significant differences in 6MWD, the mMRC dyspnea scale, SGRQ, and HADS between the intervention and control groups in both the main analysis set and PP set (Tables2  3). Assuming a clinically minimal effective change of 25 m in 6MWD, only 7 out of 41 participants among the intervention group achieved the minimal clinically important differences after the rehabilitation program.

None of the participants required hospitalization or emergency room visits related to their underlying respiratory disease or rehabilitation program during the study period. Regarding the service satisfaction questionnaire, more than half of the intervention group rated scores ≥17 (4‐8: 1 participant; 9‐12: 6 participants; 13‐16: 11 participants; 17‐20: 23 participants).

**Table 2.** Comparison of clinical outcomes of study participants at baseline and follow-up (main analysis).

Variables	Total (n=88)	Intervention (n=41)	Control (n=47)	*P* value
Baseline, median (IQR)				
mMRC^a^ dyspnea scale	1.0 (1.0‐2.0)	1.0 (1.0‐2.0)	1.0 (1.0‐1.8)	.40
6MWD^b^ (m)	483.5 (444.0‐520.0)	495.0 (460.0‐541.3)	480.0 (440.0‐503.8)	.10
HADS^c^	9.0 (6.0‐14.0)	10.0 (7.0‐14.3)	8.5 (5.0‐14.0)	.16
SGRQ^d^
Total	30.3 (20.1‐39.6)	26.5 (18.8‐36.5)	32.8 (20.6‐41.2)	.18
Symptoms	44.1 (33.5‐54.9)	41.7 (30.1‐53.8)	48.5 (33.6‐57.5)	.13
Activity	45.2 (32.2‐57.9)	44.4 (30.5‐53.9)	50.7 (32.4‐63.5)	.24
Impacts	15.5 (7.3‐28.3)	13.0 (5.9‐25.8)	18.3 (8.3‐30.7)	.26
Follow-up, median (IQR)				
mMRC dyspnea scale	1.0 (1.0‐1.0)	1.0 (1.0‐1.0)	1.0 (1.0‐1.0)	.35
6MWD (m)	485.0 (460.0‐544.0)	490.0 (468.8‐556.3)	485.0 (440.0‐527.3)	.35
HADS	10.0 (5.0‐14.0)	10.0 (5.0‐13.3)	10.0 (4.3‐15.0)	.70
SGRQ
Total	30.9 (18.5‐44.6)	27.9 (17.6‐43.7)	33.5 (22.1‐44.8)	.21
Symptoms	44.8 (32.4‐61.2)	44.9 (25.2‐54.1)	44.6 (33.2‐65.4)	.34
Activity	50.7 (31.5‐64.0)	44.4 (25.7‐57.8)	51.6 (40.1‐64.9)	.12
Impacts	16.9 (5.9‐30.1)	13.8 (5.1‐27.2)	18.0 (7.8‐31.2)	.34

a mMRC: modified Medical Research Council.

b 6MWD: 6-minute walk test distance.

c HADS: Hospital Anxiety and Depression Scale.

d SGRQ: St George’s Respiratory Questionnaire.

**Table 3.** Comparison of clinical outcomes of study participants at baseline and follow-up (compliance ≥70%, per-protocol analysis).

Variables	Total (n=62)	Intervention (n=15)	Control (n=47)	*P* value
Baseline, median (IQR)				
mMRC^a^ dyspnea scale	1.0 (1.0‐2.0)	1.0 (1.0‐1.8)	1.0 (1.0‐1.8)	>.99
6MWD^b^ (m)	480.0 (440.0‐520.0)	480 (421.3‐551.8)	480.0 (440.0‐503.8)	.65
HADS^c^	9.0 (5.8‐14.0)	9.0 (6.3‐16.0)	8.5 (5.0‐14.0)	.54
SGRQ^d^
Total	31.4 (20.2‐39.5)	26.5 (19.5‐36.0)	32.8 (20.6‐41.2)	.26
Symptoms	46.8 (33.5‐54.1)	38.0 (19.5‐53.4)	48.5 (33.6‐57.5)	.16
Activity	50.7 (33.0‐58.6)	44.4 (44.4‐56.2)	50.7 (32.4‐63.5)	.66
Impacts	16.3 (7.8‐30.4)	12.1 (6.1‐17.5)	18.3 (8.3‐30.7)	.23
Follow-up, median (IQR)				
mMRC dyspnea scale	1.0 (1.0‐1.0)	1.0 (1.0‐1.0)	1.0 (1.0‐1.0)	.29
6MWD (m)	485.0 (440.0‐538.0)	485.0 (463.3‐558.8)	485.0 (440.0‐527.3)	.54
HADS	10.5 (5.0‐15.0)	11.0 (5.0‐15.0)	10.0 (4.3‐15.0)	.90
SGRQ
Total	31.2 (18.5‐44.8)	27.9 (15.2‐42.4)	33.5 (22.1‐44.8)	.22
Symptoms	41.0 (30.6‐62.8)	36.8 (21.2‐53.1)	44.6 (33.2‐65.4)	.12
Activity	51.4 (33.1‐64.1)	44.4 (22.6‐56.2)	51.6 (40.1‐64.9)	.13
Impacts	17.5 (6.7‐31.5)	12.8 (4.3‐33.7)	18.0 (7.8‐31.2)	.44

a mMRC: modified Medical Research Council.

b 6MWD: 6-minute walk test distance.

c HADS: Hospital Anxiety and Depression Scale.

d SGRQ: St George’s Respiratory Questionnaire.

## Discussion

### Principal Findings

In this study, we evaluated the effectiveness of smartphone app–guided pulmonary rehabilitation in patients with chronic respiratory diseases. These results did not indicate a significant improvement in clinical outcomes through the use of the smartphone app–guided pulmonary rehabilitation program. Nevertheless, no rehabilitation-related musculoskeletal injuries, hospitalizations, or emergency room visits were recorded during the study period. Taken together, these results suggest that smartphone app–guided pulmonary rehabilitation may be feasible for older adults with chronic respiratory disease. Further study will be needed to clarify the clinical efficacy of this approach.

### Clinical Usefulness of Pulmonary Rehabilitation

Improvements in exercise capacity and physical activity are associated with favorable clinical outcomes in patients with chronic respiratory diseases [5,25]. Exercise capacity has been found to be a significant predictor of mortality in patients with COPD [5]. In addition, low levels of physical activity were associated with increased risks for acute exacerbation or mortality in patients with COPD [6]. In addition, the BODE index, which includes BMI, airflow limitation (FEV1), dyspnea severity (measured by the mMRC dyspnea scale), and exercise capacity (6MWD), serves as a robust predictor of mortality in patients with COPD [26]. Furthermore, disease-specific health status and quality of life, measured by SGRQ, predicted disease exacerbation and mortality in patients with COPD [27]. Anxiety or depression, as measured using HADS, has been associated with increased mortality in patients with COPD [28]. Furthermore, positive changes in HADS scores persisted even one year after the 6-week rehabilitation program for patients with COPD [29]. Therefore, although our findings did not demonstrate significant improvements in clinical outcomes, including 6MWD, SGRQ, and HADS, these findings indicate that a vigorous rehabilitation program should be recommended for patients with chronic respiratory diseases, pending further investigation [3,4,8,30].

### Compliance With Rehabilitation Programs

Previous studies have emphasized compliance with rehabilitation programs in home-based and smartphone app–based pulmonary rehabilitation. Participants who demonstrated good compliance significantly improved clinical parameters [16,31,32]. Specifically, a previous study estimated a decrease of −0.22 (95% CI −0.74 to 0.31) in the COPD assessment test for every 7-day increase in app use during pulmonary rehabilitation [33]. However, another study also indicated that the number of smartphone app users declined as the study progressed [34]. In our study, approximately one-third of participants completed exercise programs at least ≥70% of the scheduled sessions. Regarding the lack of motivation being a key factor for poor compliance in home-based pulmonary rehabilitation [35], physicians should consistently educate patients on maintaining steady rehabilitation practices during routine clinical care. Moreover, text messages or telephone contacts from health care providers may enhance motivation [36].

### Strengths

Despite most of the participants being older adults, many of them expressed satisfaction with the app and exercise program. Furthermore, we did not observe any health care usage or musculoskeletal injuries related to the rehabilitation program during the study period. These findings support the feasibility of applying smartphone app–guided pulmonary rehabilitation to older adults with chronic respiratory diseases.

### Limitations and Future Perspectives

This study also has limitations. First, although we designed the app to measure daily step counts using a smartphone-mounted pedometer, technical issues prevented us from obtaining these data. Because exercise capacity, represented by physical activity, is related to the prognosis of patients with chronic respiratory diseases (such as COPD) [5,6], further app development is necessary. Second, the participants’ preserved exercise capacity at baseline and the inherent limitations of the 6MWT may have contributed to the failure to achieve the primary outcome of improving the 6MWD. A previous study reported that healthy participants aged 60‐69 years had a 6MWD of 559 (80) meters, while those aged 70‐80 had a 6MWD of 514 (71) meters [37]. Our study participants had a median age of 68.0 (IQR 62.0‐73.0) years, and their baseline 6MWD was a median of 483.5 meters (IQR 444.0‐520.0), suggesting minimal impairments in exercise capacity. Notably, the 6MWT may have a “ceiling effect,” making it less responsive than the incremental shuttle walk test for detecting changes in COPD patients with preserved exercise capacity [38,39]. Similarly, clinical trials in pulmonary arterial hypertension, which traditionally use 6MWD as the primary endpoint, have excluded patients with preserved exercise capacity (eg, baseline 6MWD >450 m) [40]. Therefore, we recommend that future research consider participants’ baseline exercise capacity during recruitment. Moreover, the incremental shuttle walk test or cardiopulmonary exercise test should be considered for a more accurate assessment of exercise capacity [31,41,42]. Third, although we did not observe health care usage during the 12-week study period, long-term follow-up after rehabilitation may reveal the impact on disease progression or mortality. Finally, participants who dropped out after the enrollment did not undergo the follow-up measurements for the study outcomes. Thus, an intention-to-treat analysis could not be performed.

### Conclusions

In conclusion, this study highlighted a lack of improvement in clinical outcomes using smartphone app–guided pulmonary rehabilitation. However, the results did indicate that older adults with chronic respiratory conditions can safely use smartphone app–guided pulmonary rehabilitation. Thus, smartphone app–guided pulmonary rehabilitation may be feasible for older adults with chronic respiratory disease.

## Supplementary material

10.2196/64884 Checklist 1 CONSORT-eHEALTH checklist (V1.6.1).

## Abbreviations

6MWD: 6-minute walk test distance
6MWT: 6-minute walk test
CONSORT-EHEALTH: Consolidated Standards of Reporting Trials of Electronic and Mobile Health Applications and Online Telehealth
COPD: chronic obstructive lung disease
FEV1: forced expiratory volume in 1 second
FVC: forced vital capacity
HADS: Hospital Anxiety and Depression Scale
mMRC: modified Medical Research Council Dyspnea Scale
PP: per-protocol analysis
SGRQ: St. George’s Respiratory Questionnaire
